# A Glimpse of the First Eight Months of the COVID-19 Literature on Microsoft Academic Graph: Themes, Citation Contexts, and Uncertainties

**DOI:** 10.3389/frma.2020.607286

**Published:** 2020-12-23

**Authors:** Chaomei Chen

**Affiliations:** College of Computing and Informatics, Drexel University, Philadelphia, PA, United States

**Keywords:** scientometrics, visual analytics, epistemic uncertainty, citation context analysis, CiteSpace, Microsoft Academic Services, COVID-19

## Abstract

As scientists worldwide search for answers to the overwhelmingly unknown behind the deadly pandemic, the literature concerning COVID-19 has been growing exponentially. Keeping abreast of the body of literature at such a rapidly advancing pace poses significant challenges not only to active researchers but also to society as a whole. Although numerous data resources have been made openly available, the analytic and synthetic process that is essential in effectively navigating through the vast amount of information with heightened levels of uncertainty remains a significant bottleneck. We introduce a generic method that facilitates the data collection and sense-making process when dealing with a rapidly growing landscape of a research domain such as COVID-19 at multiple levels of granularity. The method integrates the analysis of structural and temporal patterns in scholarly publications with the delineation of thematic concentrations and the types of uncertainties that may offer additional insights into the complexity of the unknown. We demonstrate the application of the method in a study of the COVID-19 literature.

## Introduction

The COVID-19 pandemic has impacted so many people’s everyday life worldwide and it is still threatening our society as a whole. The COVID-19 pandemic is unprecedented in several ways that make it particularly challenging and threatening: it is still largely unknown of its origin and transmission routes; there may be months or even longer before COVID-19 vaccines can be expected to be a powerful line of defense; and the prolonged pandemic continues to pose social, economic, and political challenges to businesses and entire industries such as airlines and international travel as well as schools and many other areas.

Scientists and researchers have actively responded to the urgency and severity of the pandemic. Publications relevant to COVID-19 have increased rapidly across disciplines since the beginning of 2020. Several institutions and corporate organizations have contributed openly accessible datasets of COVID-19 publications, notably including the CORD-19 dataset and the Lens.

The COVID-19 Open Research Dataset (CORD-19) covers the scholarly literature of COVID-19, SARS-CoV-2, and the coronavirus group^1^. Its initial release contained over 29,000 articles, over 13,000 of which contained the full text. The CORD-19 dataset has been updated regularly. By the beginning of September 2020, the CORD-19 dataset reached 130,000 articles. It has been studied by many researchers, especially from communities that are well-equipped to analyze and model text documents, including data science and AI for example. A scientometric researcher, however, may find the CORD-19 dataset and other similar datasets inadequate due to the lack of cited references as part of the dataset, missing elements such as abstracts, or other data coverage or quality issues.

Several widely used analytic methods in the field of scientometrics rely on citations in scholarly publications to derive indicators and metrics of underlying research and its impact, e.g., including the h-index and the g-index. Eugene Garfield pioneered the notion of the Science Citation Index (SCI) in the 1950s, which subsequently formed the basis for citation analysis and quantitative studies of science in general (Garfield, 1955). Typically, authors of a scholarly publication refer to, i.e., cite, previously published works in their narratives. There are many studies of how and why authors ought to cite their references appropriately and how and why they often fail to do so (Greenberg, 2009). The Web of Science, Scopus, Dimensions, the Lens, and Microsoft Academic (MA) are among the most widely used multi-disciplinary bibliographic databases (Visser et al., 2020), although they differ considerably in terms of the breadth and depth of their coverage, data quality, and accessibility. Document Co-Citation Analysis (DCA) (Small, 1973) and Author Co-Citation Analysis (ACA) (White and McCain, 1998) are network-based analytic methods for studying scientific literature. Networks in such studies are constructed based on the co-occurrences of a pair of entities such as cited references or cited authors. The resultant networks then lend themselves to a wide variety of network analysis, modeling, and visualization techniques. Although networks can be derived from co-occurrences of words and phrases in scholarly publications, references may play valuable roles at a higher level of abstraction, especially in what is known as concept symbols (Small, 1978; Small, 1986). A major advantage of bibliographic databases such as the Web of Science and Scopus is the provision of references cited by a scholarly publication. These databases are conveniently used by researchers to conduct citation analysis and other citation-based studies. In contrast, PubMed, a well-known resource of the literature of biomedicine, does not provide references of an article as part of its metadata. CORD-19 provides a similar depth of coverage as PubMed, i.e.. the references cited by an article are not readily available from the dataset, which prevent researchers from conducting citation-based network analyses.

Microsoft Academic (MA) is a major source behind the construction and dissemination of CORD-19. Microsoft Academic Graph (MAG) organizes entities and relations of scholarly publications as a graph and allows flexible ways to retrieve bibliographic data, including references cited by an article (Wang et al., 2019). Furthermore, instances of a citation of a reference, often known as citances (Nakov et al., 2004), are also retrievable from MAG in terms of citation contexts. Citation contexts of a reference consist of sentences in which the reference is cited along with surrounding sentences to provide a meaningful context.

The rapid growth of the COVID-19 research literature has also led to an increasing number of bibliometric and scientometric studies, e.g., Chahrour et al. (2020), Colavizza et al. (2020), and Deng et al. (2020), as well as systematic reviews and meta-analyses of various aspects of the COVID-19 pandemic such as risk factors of critical and mortal COVID-19 cases (Zheng et al., 2020) and whether patients with asthma are at high risk (Morais-Almeida et al., 2020). Our research differs from other relevant studies of the COVID-19 literature in terms of our contribution to the development of a flexible and extensible visual analytic framework. Our goal is to empower individual researchers so that they can study a rapidly growing body of scientific literature by constructing a representative dataset of their specific interest with their own queries. Furthermore, we demonstrate how various exploratory and analytic workflows can be integrated cohesively within the visual analytic framework.

In this article, we will introduce a generic and reproducible method based on MA for visual analytic studies of the COVID-19 literature. In fact, the method is also applicable to the literature of other topics. This method contributes to the study of a research field as follows: 1) it enables anyone who is interested in the subject to construct their own CORD-19-like dataset with an enriched inclusion of cited references and citation contexts; 2) it eliminates barriers to conducting scientometric studies with citation-enriched data; 3) it expands the scope of current visual analytic studies of research literature further with the inclusion of citation contexts and enhances network-based analyses with deeper insights identified at a finer level of granularity; 4) it enables analysts to interactively explore the uncertainties associated with the citations of a reference; and 5) by conducting analyses in an integrated visual analytic environment, it enhances the implementation of a widely used science mapping tool, CiteSpace (Chen, 2004; Chen, 2006; Chen et al., 2010; Chen, 2017).

### CiteSpace

CiteSpace is a tool designed for conducting a visual analytic study of the scholarly literature of a research field, a research area, or a discipline, collectively known as a knowledge domain (Chen, 2004; Chen 2006). CiteSpace constructs a series of networks of underlying entities and their relations derived from a representative dataset of the corresponding knowledge domain. Structural patterns and trends are combined with temporal patterns and indicators to inform analysts of significant developments in a research field. A typical workflow divides a synthesized network into distinct clusters of entities such as cited references. The analyst would focus on the meaning of individual clusters and their interrelationships so as to uncover critical insights from high-levels of aggregation to lower levels. For s, applications of CiteSpace typically aim to address the following questions:

• What are the major thematic concentrations in the field of research in terms of clusters of co-occurring references?
• How are neighboring clusters connected? Which articles serve as bridges between distinct clusters?
• Which articles are the most representative members of a particular cluster?
• What would be the most accurate word or phrase to summarizes the role of a cluster?

Technically, CiteSpace decomposes a network into distinct clusters. Each cluster consists of a set of references that are frequently cited together by the same citing articles. In other words, the member references of a cluster are more often cited within the same article than references from different clusters. The quality of the decomposition is measured in terms of network modularity, the silhouette score of each cluster, and a cluster-size weighted silhouette mean. The closer these values are to 1.0, the higher the overall clarity of the configuration.

Conceptually, the position of a reference in the underlying network representation can be used to guide our navigation. According to the Structural Hole Theory (Burt, 2004), nodes located at structural holes tend to be associated with creativity, originality, and boundary-spanning. In CiteSpace, structural holes are highlighted as nodes with a high betweenness centrality score. Landmarks provide useful guidance for navigation. Highly cited articles are depicted as large tree-rings of citations per year. Articles with sharp increases in citations experience citation bursts, which indicates their particular noteworthiness because they attract extraordinary attention.

CiteSpace supports Structural Variation Analysis (SVA), which is a predictive analytic process that can be used to identify newly published articles with transformative potentials (Chen, 2012). Transformative potentials are measured in terms of significant structural variations induced by newly published articles. At a given time, a newly published article contributes to the literature by connecting previously disjoint bodies of thematic concentrations. Such connections may lead to transformative changes in the underlying knowledge structure. Transformative changes are realized if the research community follows and consolidates the promising paths forged by these innovative connections. On the other hand, not all such connections lead to changes that the research community may adapt, thus they are considered as having that potential at the point of recognition rather than as actual transformative changes.

The role of uncertainties in representing scientific knowledge is proposed in our recent conceptual framework to capture the state of the art of a research field as well as to explain what drives the dynamics of underlying research activities (Chen and Song, 2017; Chen et al., 2018). Our conceptual framework of uncertainties distinguishes three major types of uncertainties that are identified from scholarly publications, namely, epistemic uncertainty (E), hedging (H), and transitional (T). Epistemic uncertainty is identified based on the presence of uncertainty cue words that indicate the unknown, incomplete, controversial, and contradictory aspects of a topic. In order to capture this type of uncertainty from a wide variety of expressions in unstructured text, we utilized a set of semantically equivalent words of epistemic uncertainties that are commonly used in scholarly publications. These words were expanded from an initial set of seed words of epistemic uncertainties through a deep learning method explained in Chen et al. (2018). Hedging is measured in terms of hedging words such as “may,”, “suggest,” and “imply.” Hedging words are essential linguistic devices to render the certainty or, rather the uncertainty, of a statement more appropriately. Recently, there is a growing interest in the connection between hedging and citations (Small, 2018; Small et al., 2019). Transitional uncertainties are measured by the presence of transitional words such as however and nonetheless, which often signify a change in an argument or the possibility of multiple alternative interpretations of a situation. Our conceptual framework is influenced by theories of scientific change from philosophical and sociological perspectives (Kuhn, 1962; Thagard, 1992; Fuchs, 1993). As a result, epistemic uncertainties are considered as the most critical of the three types of uncertainty because epistemic uncertainties have the potential to overturn or alter our current knowledge substantially once uncertainties due to conceptual conflicts and inconsistencies are resolved. In comparison, uncertainties identified in terms of hedging and transitional vocabularies are less likely to pose a challenge to the foundation of our current beliefs.

These uncertainties are measured in terms of information entropy. The level of uncertainty can be defined at several levels of granularity, ranging from the uncertainty of a sentence in the citation context of a reference to the uncertainty of a cluster of references. Given an entity X, which can be a sentence or a set of sentences, depending on the chosen level of granularity, its uncertainty of type U, *u*_*U*_ (X), is defined as the information entropy with reference to the probability *p* (*w*_U_) of each of the uncertainty cue words, *w*_U_, of a specific type of uncertainty. The probability is estimated based on the rate of its occurrences in the entire collection of scholarly publications on Dimensions. We adopted the semantically equivalent uncertainty cue words identified in (Chen et al., 2018).

$$u_{U}\left(X\right)=-\sum_{w_{U}\epsilon X}p\left(w_{U}\right)\cdot \text{log}\left(p\left(w_{U}\right)\right)$$

Using information entropy has several advantages over other metrics. First, information entropy has been widely used to represent uncertainties. Second, the measurement of uncertainties at different levels of aggregation can be consistently defined based on information entropy. For example, the epistemic uncertainty associated with an article *u*_E_ (*r*) can be defined as the sum of all the uncertainties associated with *x*—the text passages of the article *r*, such as its citation contexts. Similarly, the uncertainty of a cluster of cited references *u*_E_ (C_k_) is naturally the sum of the uncertainties of all its member references.

$$u_{E}\left(C_{k}\right)=\underset{r\epsilon C_{k}}{\sum }u_{E}\left(r\right)=\underset{r\epsilon C_{k}}{\sum }u_{E}\left(\sum_{x\epsilon r}u_{E}\left(x\right)\right)$$

Currently, citation contexts are not available from the most commonly used bibliographic databases, such as the Web of Science, Scopus, Dimensions, and the Lens, except Microsoft Academic Services (MAS) (Sinha et al., 2015; Wang et al., 2019).

### Microsoft Academic Services

Microsoft Academic Services (MAS) provides two ways to access the Microsoft Academic Graph (MAG), either through its API to access the MAG hosted by MAS or maintaining a user’s own copy of MAG. The potential and limitations of Microsoft Academic (MA) for citation analysis have been studied and compared with other commonly used resources (Hug and Brandle, 2017; Hug et al., 2017; Thelwall, 2017; Thelwall, 2018; Visser et al., 2020). More recent reviews of MAS are presented in a study by Wang et al. (2019). An interesting study of the core concepts of classic philosophical books also made good use of the MAG (Bornmann et al., 2020).

Each article in MAG has a MAG ID, for example, 2160441315 for (Chen, 2004) and 2150999626 for (Chen, 2006). One can retrieve publications from MAG based on their MAG IDs, DOIs, and fields of study. It is also possible to retrieve articles that cite a set of references in their MAG IDs, thus this function enables us to enrich an article’s metadata with its references. For each returned article, citation contexts provide a unique advantage of MAS over other major bibliographic databases.

The integration of visual analytics of citation contexts with other citation-based analytic processes is a significant contribution of our new method. To demonstrate the application of this integrated and enhanced method, we focus on the COVID-19 literature. Instead of using the currently available CORD-19 dataset, which does not include cited references, we construct a dataset of the COVID-19 literature directly from MAS. A major advantage of this approach is its flexibility and extensibility. Anyone who is interested would be able to perform the construction with the new version of CiteSpace on a subject of their own interest. In particular, it is possible to further extend CiteSpace to support Cascading Citation Expansion (Chen and Song, 2019), which is so far only feasible in CiteSpace with Dimensions’ API. The Lens API does not currently support the retrieval of articles based on their references, although the Lens ScholarlyWorks API is very powerful in other aspects and is supported in CiteSpace.

We constructed the MA-based COVID-19 dataset with queries based on fields of study. The following query means a qualified record should match with at least one of the fields of study.

expr=Or

(Composite (F.FN==‘coronavirus disease 2019'),

Composite (F.FN==‘severe acute respiratory syndrome coronavirus 2'),

Composite (F.FN==‘2019 20 coronavirus outbreak'),

Composite (F.FN==‘covid-19'),

Composite (F.FN==‘covid19'))

The dataset was constructed on September 5, 2020. It contains a total of 79,476 records, 7,693 of which had corresponding citation contexts. This dataset represents a snapshot of the COVID-19 literature published in the first eight months of 2020 on MAG. Of these, 77,897 records have been cited at least once. These records are stored in a format that is a superset of the Web of Science field-delimited data format. The citation contexts are stored in a separate file to maximize interoperability (See Supplementary Material). The integrated analytic functions involving citation contexts use a local database. Users who do not install such databases can still use CiteSpace to process the dataset retrieved from MAS as if they were from the Web of Science.

The MAS-enriched COVID-19 dataset can be studied at three levels. First, at Level 1, the dataset contains cited references in a way similar to the bibliographic records from the Web of Science. Visual analytic studies of Level 1 datasets are increasingly common with the support of scientometric tools such as CiteSpace. All the visual analytic functions in CiteSpace that can be applied to the Web of Science data now can be applied to the MAS-enriched dataset. We will illustrate a visualization of the co-citation network with monthly time-sliced intervals and an overview of its major thematic concentrations (i.e., clusters of co-cited references). Second, Level 2, which enriches the Level-1 data with citation contexts and enables more in-depth analyses of the scholarly impact of an article at a finer granular level. Citation contexts serve as the source of information from which three types of uncertainties are measured. One reference can be cited by the same citing article in multiple citation contexts as well as by multiple citing articles in an even larger number of citation contexts (Ding et al., 2013; Hu et al., 2013). Making sense of key themes in the potentially diverse and extensive narratives is a time consuming and cognitively demanding task. Uncertainty scores provide an extra layer of metrics to highlight potentially critical information in the sense-making process. Finally, at Level 3, we demonstrate the application of the predictive SVA procedure to the dataset and identify a set of newly published articles that may be too young to stand out in terms of their citation counts but start to show signs of transformative potential.

### Data

To demonstrate the method, we constructed a dataset of the COVID-19 literature from the MAG based on matching fields of study. The resultant dataset contains publications ranging from 2014 to 2021. Interestingly, the dataset also contains publications from the future, all the way from next month to next year. Table 1 summarizes the dataset. Our subsequent discussions will focus on the subset of the first 8 months in 2020.

**TABLE 1:** A self-constructed dataset of the COVID-19 literature on Microsoft Academic Graph as of September 5, 2020.

Time of publication	Unique citing articles	Unique references	Citation contexts	Unique contexts
2014-SEP	1	14	38	23
2018-FEB	1	57	92	59
2018-NOV	1	18	32	26
2019-JAN	2	18	22	14
2019-JUN	1	5	15	15
2019-MAR	1	81	172	73
2019-MAY	1	246	443	221
2019-SEP	1	10	17	17
2019-DEC	4	102	157	96
2020-JAN	241	1,939	3,476	2,529
2020-FEB	241	1,601	3,287	2,251
2020-MAR	997	5,704	13,206	9,596
2020-APR	2,765	15,478	36,599	26,507
2020-MAY	1,657	11,227	23,131	17,089
2020-JUN	756	6,445	12,144	9,260
2020-JUL	687	6,218	11,837	8,640
2020-AUG	300	3,462	5,845	4,304
2020-SEP	18	230	371	292
2020-OCT	8	193	287	231
2020-NOV	5	76	138	107
2020-DEC	3	17	24	22
2021-JAN	2	20	27	23

Table 2 lists the number of articles found on major bibliographic databases on September 5, 2020, with the identical or closely matched queries to the query used for this study. For example, the following query was used to search in the titles only in Dimensions DSL. For full text search, replace the scope title_only with full_data.

**TABLE 2:** A simple comparison between different data sources on the search query used for this study as of September 5, 2020.

Data source	Articles	% Of CORD-19	Search strategy
CORD-19^a^	130,000	100.00	Combined
Dimensions^b^	124,131	95.49	Full text
Lens^c^	100,759	77.51	Full text
Lens	98,839	76.03	Title, abstract, keyword
Dimensions	90,747	69.81	Title, abstract, keyword
Lens	83,048	63.88	Title
MAG^d^	80,676	62.06	Fields of study
Dimensions	75,435	58.03	Title
Google scholar^e^	73,700	56.69	Title, abstract, full text
Web of science^f^	29,858	22.97	Topic search

^a^ https://www.semanticscholar.org/cord19

^b^ https://app.dimensions.ai/discover/publication

^c^ https://www.lens.org/

^d^ https://docs.microsoft.com/en-us/academic-services/

^e^ https://scholar.google.com/schhp?hl=en

^f^ https://clarivate.com/webofsciencegroup/solutions/web-of-science/

search publications in title_only for “\”coronavirus disease 2019\” OR \”severe acute respiratory syndrome coronavirus 2\” OR \”2019 20 coronavirus outbreak\” OR \”covid-19\” OR covid19” return publications [id]

A search on Google Scholar used the following query:

‘coronavirus disease 2019' OR ‘severe acute respiratory syndrome coronavirus 2' OR ‘2019 20 coronavirus outbreak' OR ‘covid-19' OR ‘covid19'

The search on MAG used the following query:

expr=Or(Composite(F.FN==‘coronavirus disease 2019'), Composite(F.FN==‘severe acute respiratory syndrome coronavirus 2'), Composite(F.FN==‘2019 20 coronavirus outbreak'), Composite(F.FN==‘covid-19'), Composite(F.FN==‘covid19'))

The CORD-19 dataset is the most comprehensive with 130,000 articles. Full text searches on Dimensions and the Lens returned over 100,000 articles. As anticipated, searches in the metadata alone returned fewer articles than full text searches. We used fields of study to construct a demonstrative dataset to reduce the complexity of the query formation. More sophisticated strategies such as Cascading Citation Expansion can be used to optimize the overall quality of the data collection step (Chen and Song, 2019). A topic search in the Web of Science returned the lowest number of articles in this group. In terms of the percentage of the CORD-19 dataset, the Web of Science represents about 23% of its volume, MAG over 62%, and full text searchers on Dimensions and Lens over 75%.

## Overview

It is important to identify patterns and trends in the emerging literature on COVID-19. Which areas of research are particularly active in the first eight months of the pandemic?

**FIGURE 1:**
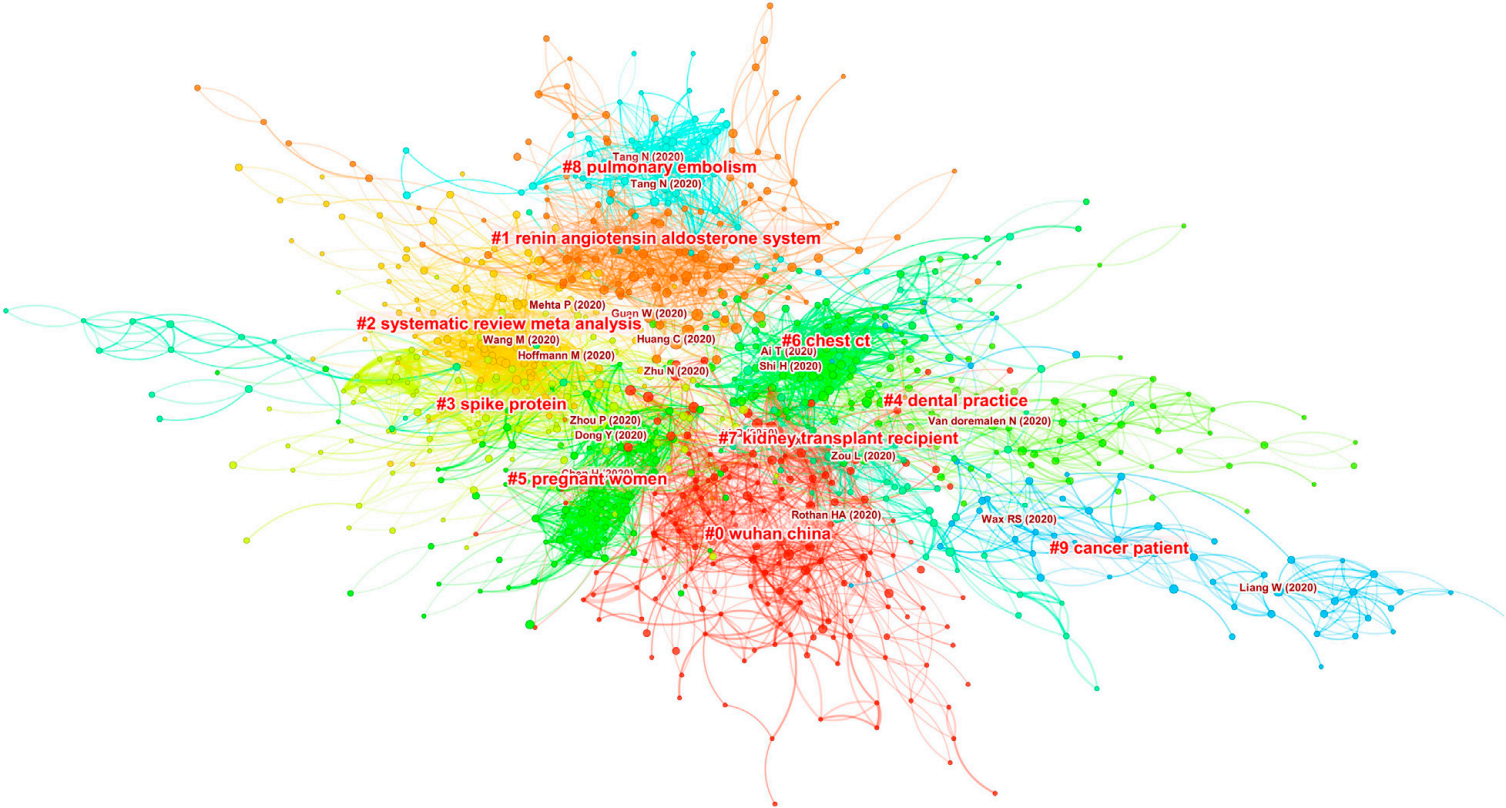
An overview of 1,330 top-cited articles in the COVID-19 literature of 77,897 articles. The size of a node represents the number of times the corresponding article has been cited in the dataset. The prominent theme of each cluster of cited articles is algorithmically labeled.

Figure 1 shows an overview of the underlying network of references that are often cited together. The nodes are references cited by citing articles, which are records from the dataset retrieved from MAG, whereas links between them represent the strengths of their co-occurrences. Groupings of references, or clusters, emerge as some references are co-cited more often than others. These clusters represent concentrations of themes, although the degrees of concentration may vary widely across different clusters. Each cluster is assigned an automatically generated cluster label, for example, #3 spike protein and #5 pregnant women. Clusters are numbered from #0 onwards. Clusters are depicted in different colors, starting with the largest cluster #0 in red and followed by smaller clusters in yellow, green, and other colors from a rainbow colormap. We are of course aware that the rainbow colormap may be harmful if used inappropriately but in this case as long as we can differentiate one cluster from another, it would be sufficient.

The largest cluster #0 Wuhan China is shown in red near the center of the network. The second largest cluster #1 renin angiotensin aldosterone system is located near the top. Clusters #2 systematic review meta analysis, #3 spike protein, #5 pregnant women can be found on the left. On the right half of the graph, there are clusters #4 dental practice, #6 chest [CT], and #12 mental health.

The configuration of the parameters for this network is shown in the upper left corner. Users may adjust them accordingly. The provision of citation contexts provides another option to enhance the conventional Document Co-Citation Analysis (DCA), which typically treats each reference with equal weight for a citing article because no further data beyond the reference list is available. In this study, we utilize the additional information accessible from the corresponding citation contexts such that references frequently cited in the article can be retained, whereas references cited below average can be discarded. Users may control this option.

### Concept Trees

The MAG-based COVID-19 dataset contains 111,360 citation contexts made by 7,693 citing articles, involving 39,995 references. Although this represents only 9.88% of the 77,897 citing articles that have at least one citation in the dataset, the available citation contexts provide enough information to assess the feasibility of the integrative approach.

The overview in Figure 1 highlights the most active areas of the literature. To understand what each cluster involves, one option is to characterize the thematic structure of the cluster in a hierarchical representation of its component concepts (Palla et al., 2015; Balogh et al., 2019). Given a set of text documents such as titles, abstracts, and citation contexts, constructing a concept tree in CiteSpace consists of the following steps:

1. Extracting noun phrases from the text preprocessed with part-of-speech tagging;
2. Deriving hierarchical relations between phrases at the sentence level such that if a noun phrase n_A_ co-occurs with noun phrases n_B_ and n_C_, then n_A_ is considered as a higher-level concept on the hierarchical structure;
3. Visualizing the hierarchical structure as a concept tree.

**FIGURE 2:**
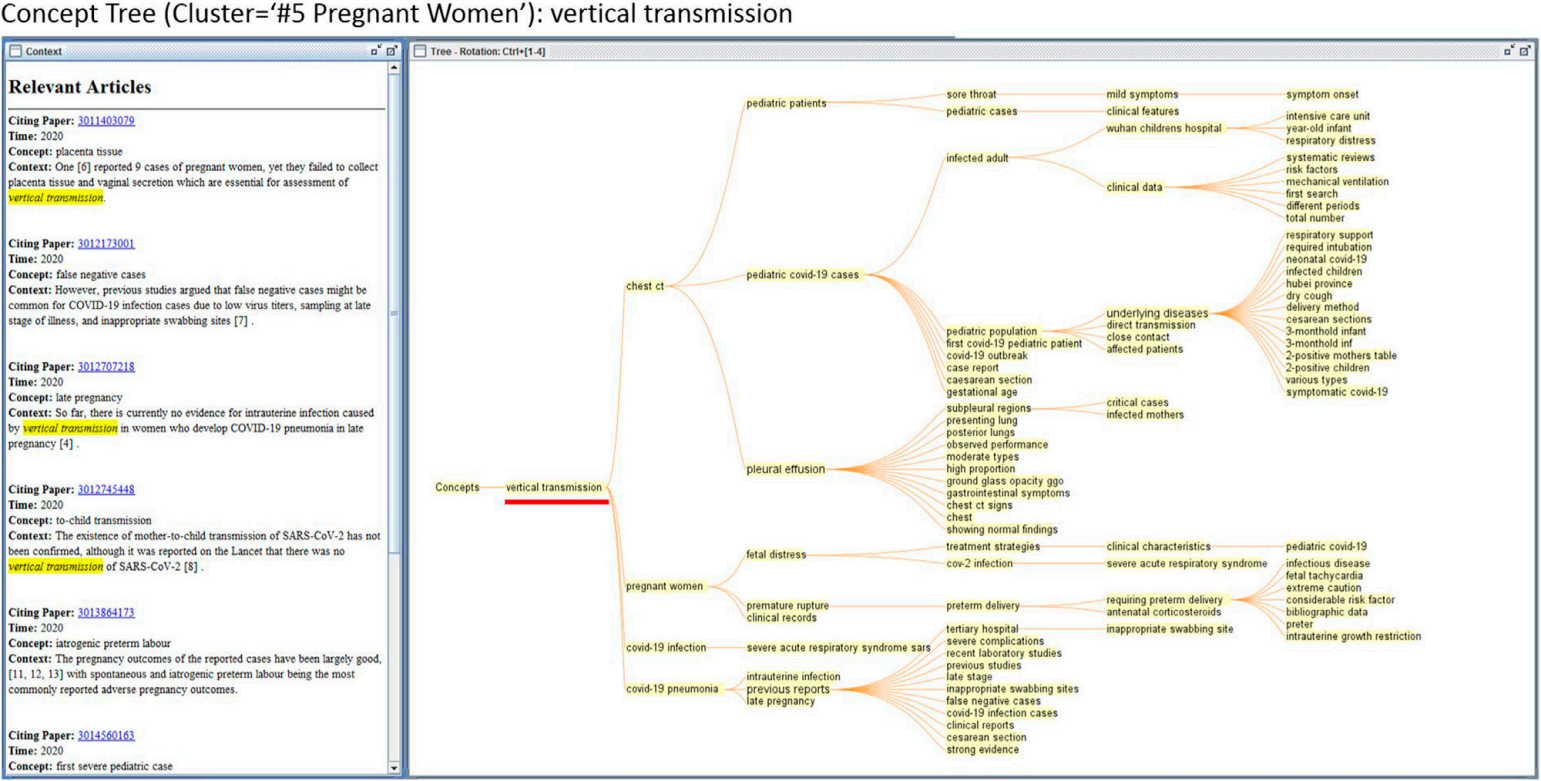
Making sense of a cluster (#5 pregnant women). The list of citation contexts shown in the left window corresponds to the current mouse-over event on the concept of vertical transmission.

Figure 2 shows a concept tree of Cluster #5 pregnant women. The nodes of the tree are concepts, which are noun phrases extracted from the citation contexts of the member references in this cluster. Major branches of the tree highlight the key themes or concerns of the cluster. For example, vertical transmission is shown as the root of the tree, which is the primary concern of this cluster. Users may interactively inspect the citation contexts of each concept, as shown in the right window in Figure 2. The citing paper’s MAG ID is shown as a hyperlink. Following the hyperlink will take the users to the record displayed on the Microsoft Academic website.

**FIGURE 3:**
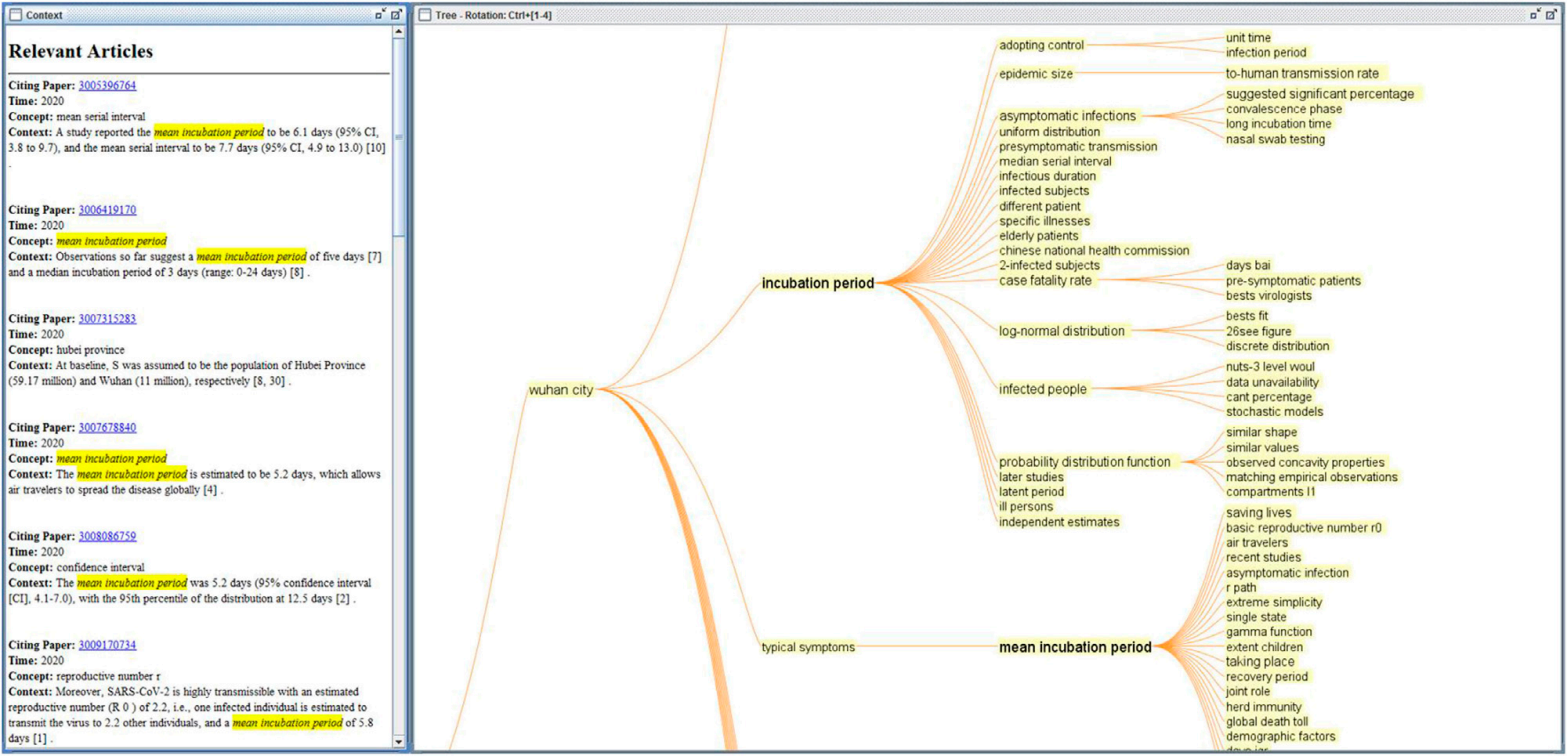
Making sense of major themes of citations to a specific reference Li Q. (2020).

Concept trees can be used to highlight the structure of a cluster, a reference, or a word or a phrase. Figure 3, for example, is generated from a single reference that has the highest epistemic uncertainty score. The concept tree reveals that the incubation period and mean incubation period are the major themes that emerged from citation contexts made by subsequently published articles. Interactive inspections of the concept of the mean incubation period reveal the specific days mentioned in various citation contexts, shown in the left window. For example, the mean incubation period of 5.2°days appears frequently in these contexts. This function can facilitate traditional systematic reviews and meta analyses as one can efficiently collect the values of a specific variable from a large number of publications.

### Uncertainties

Each citation context consists of a few sentences in which a particular reference is cited by a citing article. The uncertainties of these sentences can be measured in terms of the presence of uncertainty cue words and how often these cue words appear in a global bibliographic database such as the entire collection of over 200 million articles on Dimensions. Semantically equivalent cue words are learned from initially hand-picked cue words as seeds (Chen et al., 2018). Epistemic uncertainties are designed to capture uncertainties in scientific knowledge, which can be manifested by the appearance of cue words such as controversial, contradictory, inconsistent, unknown, and uncertain.

While the uncertainties of citation contexts can be identified independently from the underlying topic as shown in the above example, a more valuable option is to combine the level of uncertainty with specific rhetorical cues or specific concepts. For example, we may want to see how epistemic uncertainties are associated with concrete concepts such as social distancing, face masks, and international travel. As shown in Table 3, a combination of epistemic uncertainty cues and rhetorical words on conclusions reveals additional insights into the challenges we are facing.

**TABLE 3:** Epistemic uncertainties of citation contexts containing specific rhetorical words on conclusions.

Citing Article→Cited reference	Epistemic uncertainty	Citation context Uncertainty: Uncertain/conflicting/contradict/inconsistent rhetorical: Conclusion/conclude
3037877512→3018691224	0.0314	Rly complex and counter-intuitive due to the **uncertainty** in the transmission mechanisms, possible seasonal variation in both susceptibility and transmission, and their variation within subpopulations [7]. The media has given extensive coverage to analyses and forecasts using COVID-19 models, with increased attention to cases of **conflicting** *conclusions*, giving the impression that epidemiological models
3079224143→3013360115	0.0314	[35] *Conclude* that not only the COVID-19 numbers will grow but also **uncertainty** about forecasts will also grow
3020670761→3010344953	0.0314	(14) Finally, a recent systematic review of the literature *concluded* that while further research on this topic was required, the limited data available, albeit not adjusted for other factors potentially impacting on outcome, *concluded* that smoking was most likely associated with negative progression and outcomes of SARS-CoV-2, (15) while a second meta-analysis *concluded* that active smoking was not associated with severity of SARS-CoV-2.(16) Nevertheless, despite this current **uncertainty** many organisations, such as the World Health Organization and the National Institute of Drug Abuse, recommend smoking cessation strategies not only to alleviate the harm caused by smoking, in general, but also because smoking cessation may potentially lessen the risks of SARS-CoV-2 infection
3023144169→2969352266	0.0237	It can be *concluded* that the results of previous studies on the immune system have been **inconsistent**
3009935283→2811210701	0.0205	21 The **conflicting** *conclusions* from our two scenarios, driven largely by the differences in the extent of presymptomatic transmission, highlight the urgent need for more data to clarify key epidemiological parameters of COVID-19, particularly the serial interval and the extent of presymptomatic transmission, in order to inform response efforts
3021685303→2802058961	0.01	Moreover, the **contradictory** findings about smoking in the literature, with studies published before the COVID-19 pandemic reporting that smoking and nicotine down-regulate ACE2 [3, 4] and other studies published during the pandemic reporting that they up-regulate ACE2 [5] [6] [7], do not allow for solid *conclusions* regarding the effects of nicotine or smoking on ACE2
3007114958→3002108456	0.0014	Compared with the results of the two studies on Wuhan cases by Chen et al. 18 and Huang et al. 19, we found that the gender proportion was equal in the 80 patients we included, **contradicting** to the *conclusion* that men were more susceptible than women

Uncertainty cue words are in bold, whereas rhetorical words are in italic.

**FIGURE 4:**
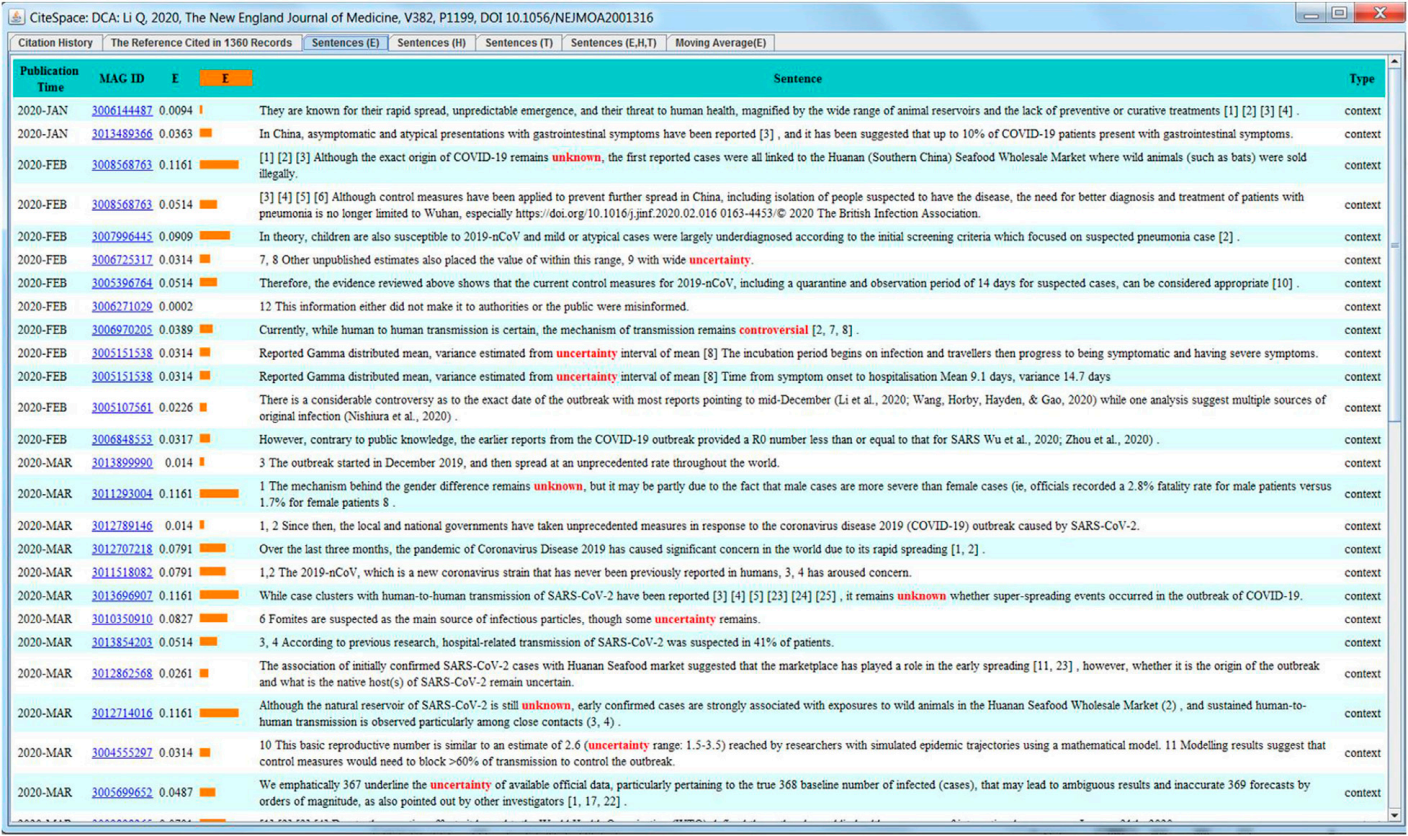
Uncertainties of citation contexts of Li et al. (2020).

Figure 4 shows a screenshot of the Node Details window in CiteSpace for the article (Li Q. et al., 2020), which has the largest epistemic uncertainty score in the dataset. The screenshot shows a list of citation contexts of the reference in chronological order. The length of an orange bar depicts the epistemic uncertainty score (E). Major uncertainty cue words contributing to the uncertainty score are highlighted in red, including unknown, uncertainty, and controversial. The visual representation reduces the cognitive burden of manually sifting through numerous citation contexts across different articles. It makes it easier for us to concentrate on key information. We can quickly scan citation contexts and focus on those with high uncertainty scores. We believe that uncertainties can provide valuable insights into the heart of research (Chen and Song, 2017).

**FIGURE 5:**
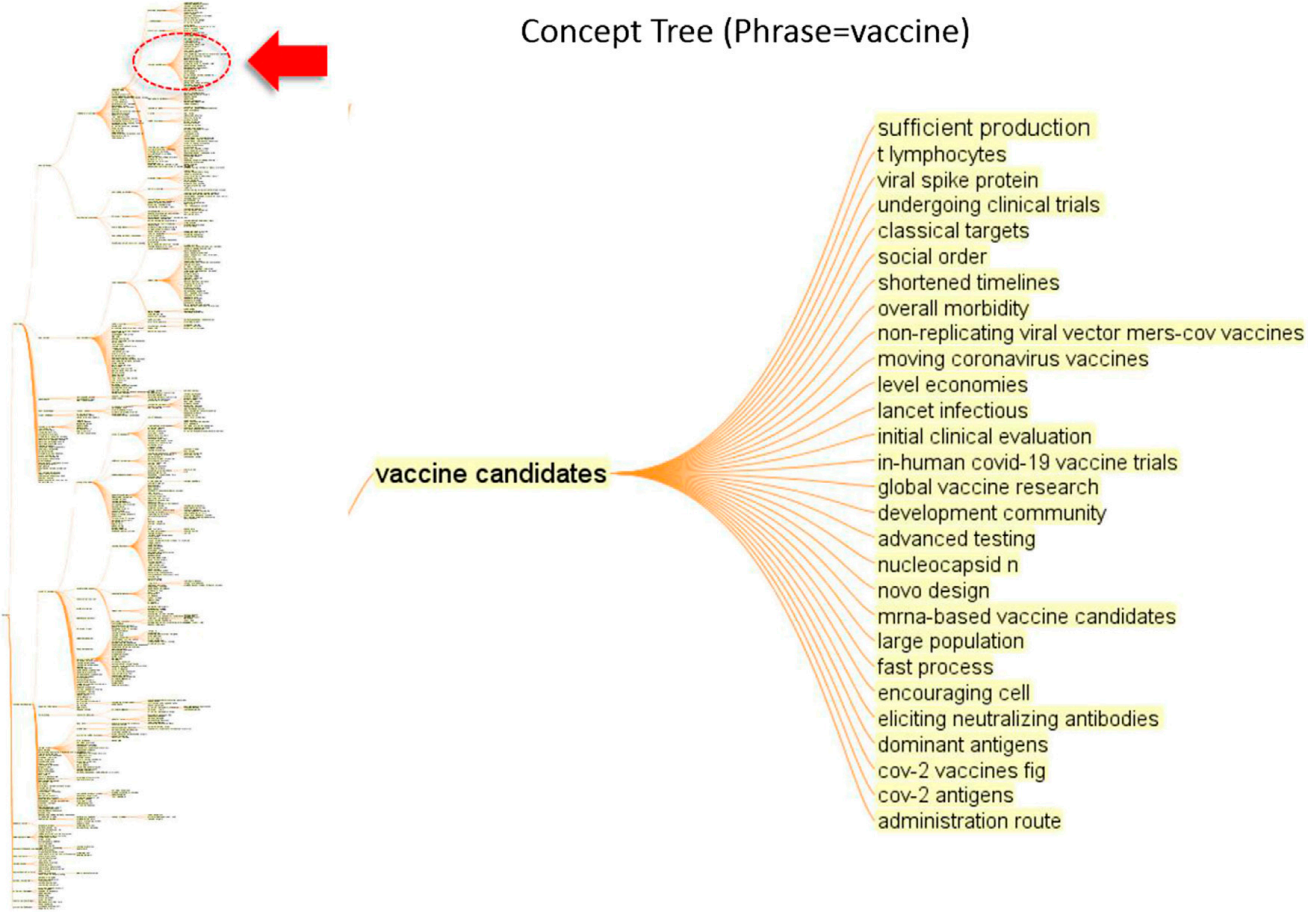
Concept tree of a phrase: vaccine.

Figure 5 shows a concept tree of the word, “vaccine.” The concept tree on the left is the entirety of the hierarchical representation of the concepts co-occurring with the word “vaccine” or “vaccination.” One can also interactively inspect other nodes in the concept tree and their citation contexts.

The examples shown above illustrate the application of the method on datasets retrieved from MAG. The following examples demonstrate Structural Variation Analysis (SVA), which is a predictive analytic method to identify newly published articles with transformative potentials (Chen, 2012). In other words, SVA aims to identify new articles that may have a profound impact on the underlying literature in the future.

### Structural Variation Analysis (SVA)

The structural variation analysis is built on the basis of Structural Hole Theory, which was originally proposed for social networks (Burt, 2004; Chen, 2012). According to the Structural Hole Theory, people’s positions in their social network or a network of their co-workers may be associated with social capital, which may be in turn translated into a competitive edge. Furthermore, positions that play a significant role in connecting different parts of the network tend to have greater potential than other positions. From an information flow point of view, individuals who are in such positions are exposed to different ideas, perspectives, and opinions, which makes them more open-minded and creative. We extended this insight to networks of scholarly publications.

References located at structural holes in a network have the potential to make a profound impact on the global structure of the underlying research field. The SVA procedure looks for newly added connections that may alter the global structure or have the potential to do so. In essence, each newly published article is compared to a baseline network formed as a snapshot of the literature prior to the publication of the article. Co-occurring links made by the article are examined in the baseline network to determine whether a specific link is transformative or incremental. Transformative links are novel connections between distinct clusters, whereas incremental links are within-cluster connections.

**FIGURE 6:**
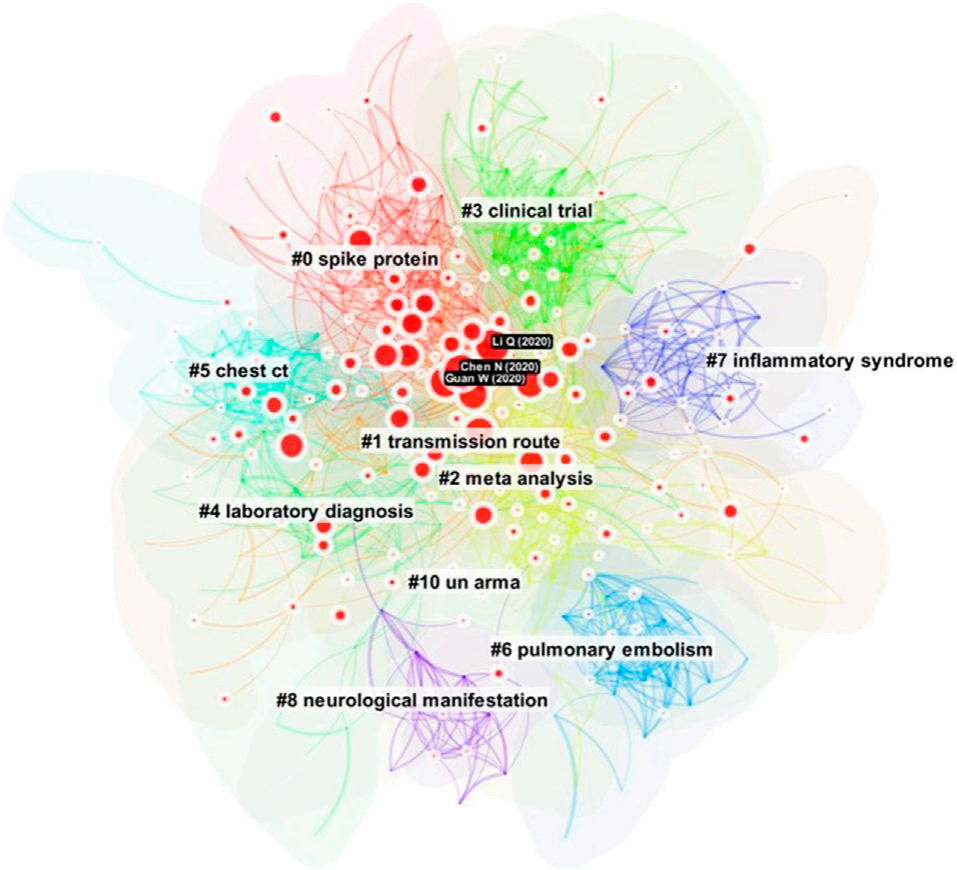
An overview of a smaller network to illustrate SVA. The size of a disc in red depicts epistemic uncertainty (E). The largest three discs are labeled with a black background.

Figure 6 shows an overview of the network we will work with for SVA. The current implementation of SVA is computationally expensive; therefore, the demonstration used a smaller network than the one discussed earlier in this article. The overview shows a similar set of clusters with slight shifts of focus. The largest cluster is #0 spike protein. The second largest one is #1 transmission route. The three articles labeled with a black background are the three articles with the highest epistemic uncertainty scores, including an article by Li Q. et al., 2020, discussed earlier in this study.

**FIGURE 7:**
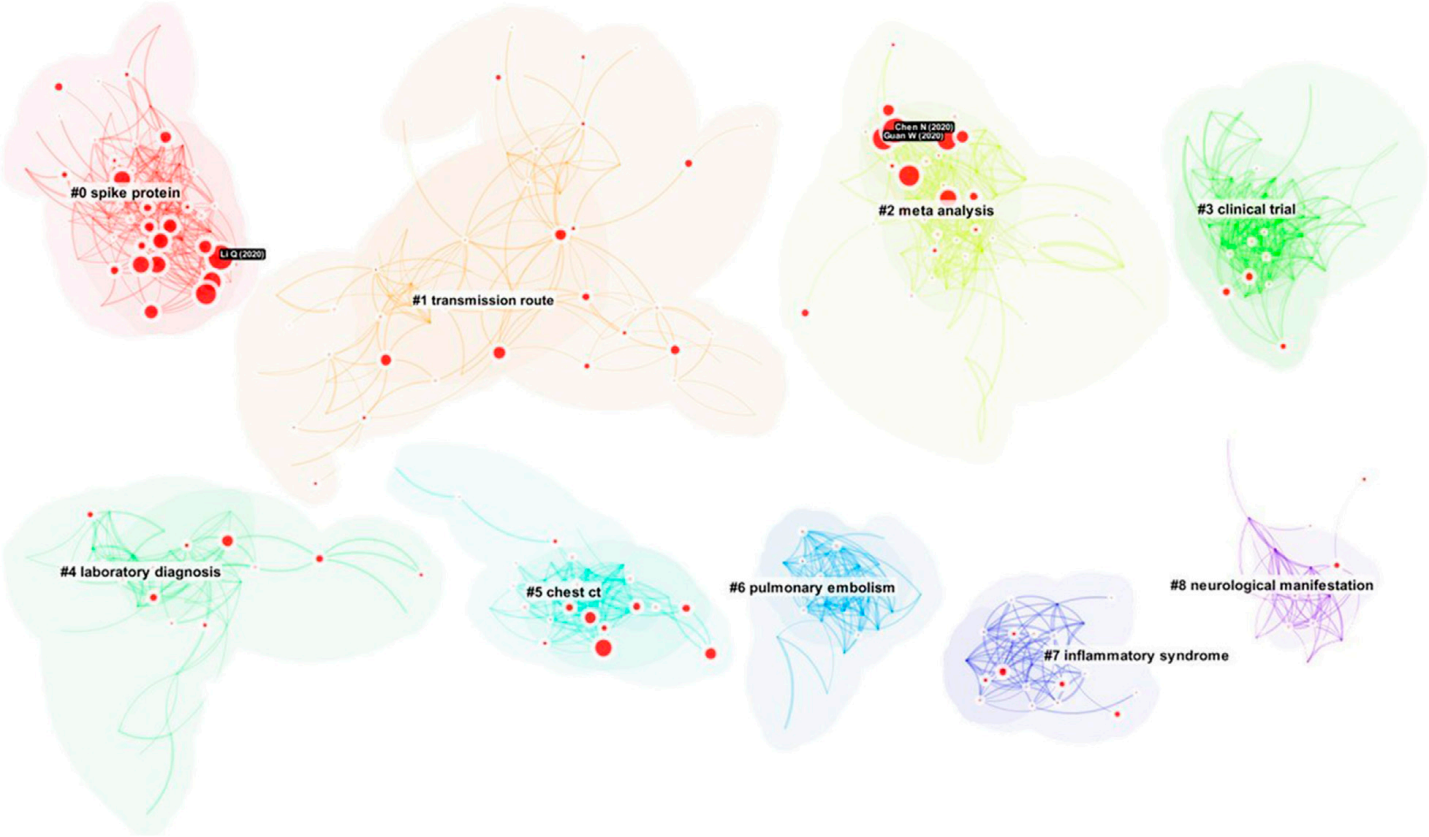
The distribution of citation contexts with uncertainties is uneven. The most uncertainties are in clusters 0, 2, and 5.

Figure 7 shows the same set of clusters in a more separated arrangement. The separation reveals that the largest cluster #0 spike protein contains the most nodes with significant levels of epistemic uncertainty. One of the three most “uncertain” nodes, (Li Q. et al., 2020), is in this cluster, whereas the other two belong to cluster #2 meta analysis. In contrast, the overall uncertainty of cluster #6 is very low. The distribution of epistemic uncertainty at the cluster level requires further investigation. One possible hypothesis is that high epistemic uncertainty indicates a sophisticated level of articulation in scholarly communication. A high concentration of articles with epistemic uncertainty may also indicate the origin of a field of research when researchers conceptualize fundamental research needs.

**FIGURE 8:**
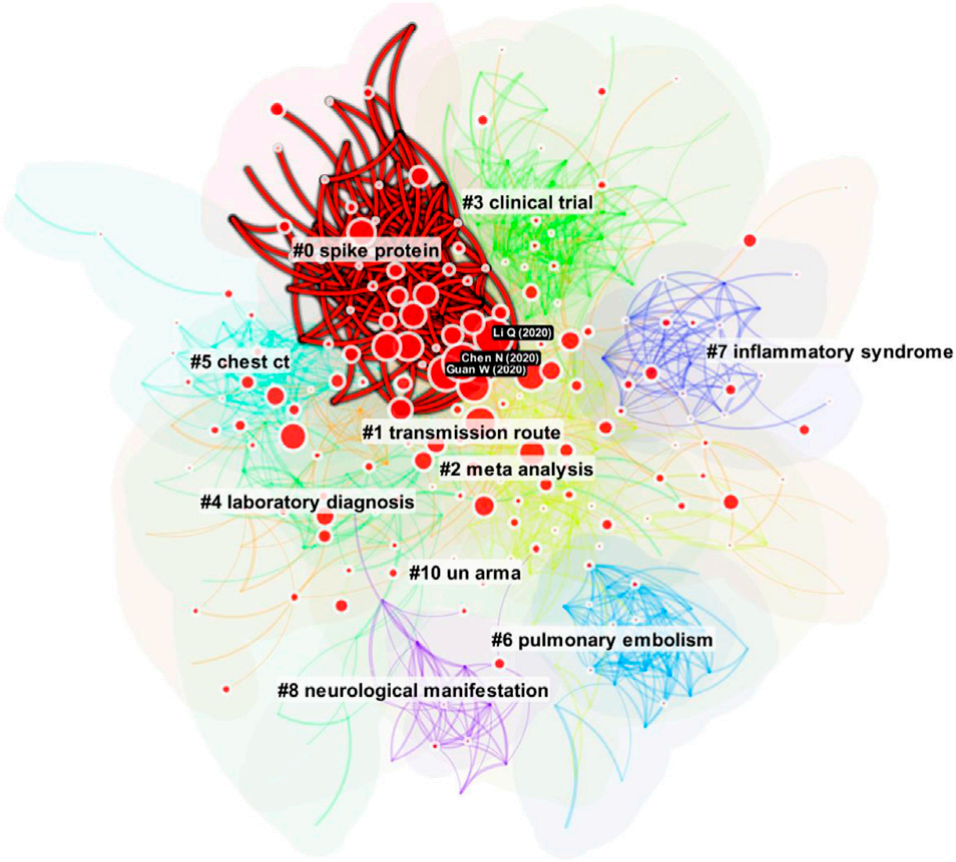
References associated with the strongest sentiment of uncertainty are from Cluster #0 spike protein.

Figure 8 shows an overlay of cluster #0 spike protein on the overall network. Such overlays highlight the scope of a cluster and inter-cluster relations. The visualization also suggests that the majority of the epistemic uncertainty appears to concentrate on the central area of the network.

**FIGURE 9:**
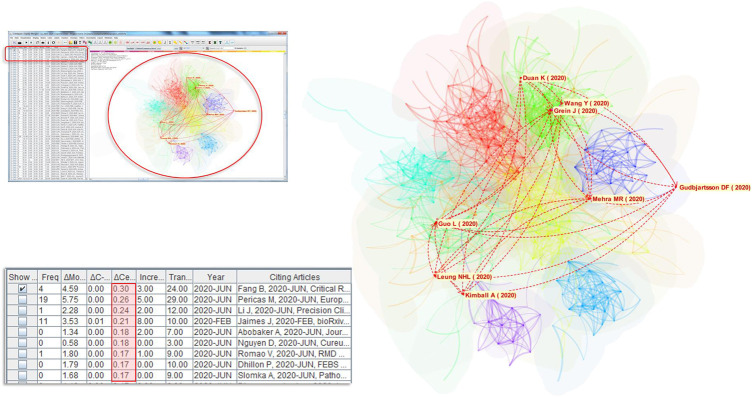
An article identified by SVA with a high transformative potential according to centrality divergence.

Figure 9 shows a newly published article identified with a high transformative potential based on the centrality divergence metric, one of the three structural variation metrics. The article is also ranked high on another metric–modularity change. The visualization reveals the distribution of the references cited by the article across different clusters. The next question is whether these cross-cluster connections reveal an underlying boundary-spanning mechanism.

The review article by Fang and Meng (2020) was published in June 2020, but its citation contexts are not found on MAG at the time of writing. We manually located the citation contexts from the original publication. Table 4 shows the citation contexts of four of the references cited by Fang and Meng (2020). Each of the references is cited in different sections on distinct topics, which partially explains the diverse footprint found by SVA.

**TABLE 4:** Citation contexts of the references cited by Fang and Meng (2020).

Cluster	References	Citation context	Section heading
#4 laboratory diagnosis	Guo et al. (2020)	Infection of SARS-CoV-2 triggers the host humoral response, leading to the generation of antibodies including IgA, IgM, and IgG against SARS-CoV-2 [**78**]	#5 Serology testing
		Another study with N protein-based ELISA on 208 plasma samples from 82 confirmed and 58 probable cases revealed that the median time for detection of IgM and IgA was 5 days while for IgG it was 14 days after symptom onset [**78**]	#5 Serology testing
#8 neurological manifestation	Kimball et al. (2020)	A PCR screening test on 78 residents in a long-term care nursing home in Washington State resulted in the detection of 10 symptomatic, 10 pre-symptomatic, and 3 asymptomatic cases [**101**]. symptomatic, 10 pre-symptomatic, and 3 asymptomatic cases [**101**]	#4 Large scale screening to detect asymptomatic or pre-symptomatic cases
#3 clinical trial	Grein et al. (2020)	Preliminary results showed that about 68% of patients with severe COVID-19 treated with compassionate-use of remdesivir had clinical improvement [**14**], whereas a randomized, double-blind, and placebo-controlled multicenter trial showed remdesivir was not associated with significant clinical benefits [15], suggesting additional efficacy studies will be needed to demonstrated remdesivir’s benefit	#1 Introduction
#7 inflammatory syndrome	Mehta et al. (2020)	Possible roles of cytokine storm syndrome that leads to critical disease and death of COVID-19 patients have been discussed [**54**,55]	#3 Clinical characteristics of COVID-19
		In fact, compared with non-intensive care unit (ICU) patients, ICU patients had higher plasma levels of IL-2, IL-6, IL-7, granulocyte-colony stimulating factor, interferon-γ inducible protein 10, monocyte chemo-attractant protein 1, macrophage inflammatory protein 1-α, and TNFα [7,48,49,**54**]	#3 Clinical characteristics of COVID-19
		In critically ill patients, cytokines and other biomarkers are significantly changed and measurement of these biochemical markers can be used to determine the severity and mortality of the disease [7,48,50,**54**]	#3 Clinical characteristics of COVID-19

A practical question in constructing a systematic review of a subject given the current landscape of its literature, is which areas are promising and might influence future directions and breakthroughs in research? In the case of the COVID-19 pandemic, monitoring the spread of the virus and the development of new vaccines are among the top of such a list. About 10% of the records in the MAG-based COVID-19 dataset have corresponding citation contexts available. Nearly 15% of them contained the words vaccine or vaccination. SVA enables researchers to watch activities in valleys and voids on the current literature landscape, allowing us to explore transformative potentials that might bring different threads of research together. The literature-based discovery community pioneered by Don Swanson has been devoted to uncovering these types of connections that might be missing or weak in the current literature (Chen and Song, 2019).

Table 5 shows a list of articles with the strongest transformative potentials in terms of modularity change, which is essentially consistent with the citation-based ranking, suggesting that the validity of the list can be further verified in the near future when their citation contexts become available. The harmonic mean of the modularity change (M), cluster linkage (C-L), and centrality divergence (C-D) further simplifies the identification of newly published articles that may have high transformative potential based on the three structural variation metrics. The NR column in the table shows the number of references cited by the corresponding article. An article with a high NR value is likely to be a systematic review of the literature, which is also more likely to demonstrate a boundary-spanning effect (Chen, 2012). Most of the articles on the list were published in June 2020, slightly over two months earlier than the time of the study. The second one on the list has already been cited 19 times. It shows how fast the COVID-19 literature is growing as well as a promising visibility of this article. The title of the article is “COVID-19: From epidemiology to treatment.” Interested readers may revisit this list regularly in the next few months and monitor whether the transformative potential is realized and strengthened or diminished and forgotten.

**TABLE 5:** Some of the articles with the strongest transformative potentials in terms of M for modularity change. C-L is for cluster linkage. C-D is for centrality divergence. The Harmonic column shows the harmonic mean of M, C-L, and C-D. The NR column is the number of references cited by the corresponding article.

MAG ID	M	C-L	C-D	Harmonic	Citations	NR	Title	References
3006967091	9.082	0.242	0.134	0.257	60	32	2019 Novel coronavirus of pneumonia in wuhan china emerging attack and management strategies	She et al. (2020)
3033364035	5.748	0.020	0.260	0.055	19	105	Covid 19 from epidemiology to treatment	Pericàs et al. (2020)
3036326077	4.594	0.016	0.303	0.047	4	116	The laboratory's role in combating covid 19	Fang and Meng (2020)
3006282354	3.531	0.105	0.209	0.206	11	57	Structural modeling of 2019 novel coronavirus ncov spike protein reveals a proteolytically sensitive activation loop as a distinguishing feature compared to sars cov and related sars like coronaviruses	Jaimes et al. (2020)
3035754070	2.984	0.010	0.138	0.029	0	80	Covid 19 and the cardiovascular system a review of current data summary of best practices outline of controversies and illustrative case reports	Prasad et al. (2020)
3028751559	2.277	0.008	0.239	0.024	1	123	The epidemiology and therapeutic options for the covid 19	Li J. et al. (2020)
3037777580	1.796	0.006	0.174	0.018	1	56	Rheumatology practice amid the covid 19 pandemic a pragmatic view	Romão et al. (2020)
3032928719	1.787	0.007	0.170	0.020	0	104	Covid 19 breakthroughs separating fact from fiction	Dhillon et al. (2020)
3036140173	1.684	0.006	0.170	0.018	0	134	Coronavirus disease 2019 covid 19 a short review on hematological manifestations	Slomka et al. (2020)
3033243614	1.338	0.005	0.176	0.014	0	51	Extrapulmonary and atypical clinical presentations of covid 19	Abobaker et al. (2020)
3036886562	1.194	0.004	0.170	0.012	0	29	Covid 19 and advanced practice registered nurses frontline update	Diez-sampedro et al. (2020)
3039476797	1.039	0.003	0.150	0.010	0	157	Covid 19 progress in diagnostics therapy and vaccination	Liu et al. (2020)
3035971111	1.012	0.003	0.148	0.010	0	42	Gastrointestinal manifestations of covid 19	Ouali (2020)
3044931581	0.789	0.003	0.137	0.008	0	45	Sars cov 2 infection in children	Çokuğraş and Önal (2020)
3036299138	0.579	0.002	0.128	0.006	1	44	Targeting cytokine storm to manage patients with covid 19 a mini review	Roshanravan et al. (2020)
3036482760	0.579	0.002	0.147	0.006	6	102	Severe covid 19 and aging are monocytes the key	Pence (2020)
3037103256	0.577	0.002	0.180	0.006	0	14	Pediatric case of severe covid 19 with shock and multisystem inflammation	Nguyen et al. (2020)
3033462763	0.357	0.001	0.132	0.004	0	46	Covid 19 pandemic and pediatric population with special References to congenital heart disease	Malviya and Yadav (2020)
3036315910	0.213	0.001	0.140	0.002	0	13	Strategies for successful catheterization laboratory recovery from the covid 19 pandemic	Poulin and Pinto (2020)
3036932243	0.200	0.001	0.147	0.002	0	219	Sars cov 2 an update on potential antivirals in light of sars cov antiviral drug discoveries	Elshabrawy (2020)

## Discussion

The examples discussed in the present study demonstrate the advantages and further potentials of integrating MAG for the study of a rapidly growing body of literature over a monthly time scale, compared to the much longer time frame typically used in contemporary scientometric studies. Our experience shows that it is feasible for end-users to construct their own datasets at a pace of their own choice. More importantly, this method opens a wide range of possibilities for researchers to compare different bibliographic databases. Until recently, such possibilities were limited to the few due to the resource-demanding nature of this type of analysis (Chen and Song, 2019; Visser et al., 2020).

The method described here demonstrates how end-users could be able to conduct scientometric studies using commonly used methods such as DCA in the same way as they would with datasets from long established but relatively more selective sources such as the Web of Science. At the same time, an equivalent topic search in the Web of Science returned 29,858 records, whereas CORD-19 has reached 130,000 records. MAG is in the same category as the Lens and Dimensions, within the range of 70,000–90,000 records.

Futhermore, integrating citation contexts with the visual analytic workflow significantly extends the depth of scientometric studies. The ability to cross-reference structural indicators and the linguistic cues of uncertainties has great potential to enrich the sense-making process of a rapidly growing body of the literature. This integrated method enables analysts to visually inspect all the instances in which the mean incubation period is discussed with reference to a particular study. The use of interactive visualization considerably reduces the cognitive load of visual exploration of scientific literature. Such improvements are promising examples of how integrating network-based visualization and analytic approaches with text-based visual exploration can be guided by different types of uncertainty metrics.

Our examples demonstrate that the integrated approach can further push the capabilities of scientometric studies and the provision of citation contexts opens up valuable opportunities for researchers to monitor and track articles with transformative potentials. It is also conceivable that one may extend studies along the direction demonstrated in (Bornmann et al., 2020), which resembles a closed search when researchers identify concepts they would like to trace, as opposed to an open-ended search when researchers would welcome concepts identified by computational models and/or interactive visual exploration.

MAG does have limitations. The most noticeable one is the relatively low rate of records with citation contexts at the time of writing. We found about 10% of the records in the dataset have citation contexts. We tested the distribution of the records with citation contexts by overlaying the network of records with citation contexts on the network of all records. The result is assuring–the records with citation contexts are usually located in the core of the COVID-19 network, whereas articles with missing citation contexts tend to be located at the peripheral of the network. Even with the current availability rate of citation contexts, our study has shown the feasibility of such studies to address specific questions such as the consensus on the mean incubation period in the literature. Encouraged by the recent review of MAS (Wang et al., 2019), we believe MAS will continue to improve and the overall quality of MAG as a data source will steadily increase. The current MAS allows free API access to the MAG with a minor compromise in terms of the limit of API calls per minute and the total calls per month. Another reason why MAG provides a promising method of facilitating visual analytic studies of scientific literature is that it allows users to host their own copy of MAG themselves, indicating that these restrictions can be eliminated.

## Conclusion

In conclusion, this study has introduced a visual analytic method that can overcome a few significant shortcomings in the practice of conducting scientometric studies of the literature. The method provides a flexible and extensible approach that allows researchers to tailor the breadth and the depth of a dataset to meet their own requirements. It enables researchers to apply existing tools to self-constructed datasets on topics of interest at a pace of their own choice. Easy access to citation contexts provides valuable contributions to the workflow of visual analytic studies of scientific literature. Integrating citation contexts with network-centric analytic paradigms may serve as a stepping stone to creating a fully integrated workflow for the study of scientific literature at all levels of detail as well as in identifying insightful patterns and promising trends.

## Data Availability Statement

The original contributions presented in the study are included in the article/Supplementary Material, further inquiries can be directed to the corresponding author.

## Author Contributions

The author confirms being the sole contributor of this work and has approved it for publication.

## Funding

National Science Foundation SMA-1633286.

## Conflict of Interest

The author declares that the research was conducted in the absence of any commercial or financial relationships that could be construed as a potential conflict of interest.

## Footnotes

1
https://www.whitehouse.gov/briefings-statements/call-action-tech-community-new-machine-readable-covid-19-dataset/
